# Implications of the Thermodynamic Response of Soil Mineralization, Respiration, and Nitrification on Soil Organic Matter Retention

**DOI:** 10.3389/fmicb.2021.651210

**Published:** 2021-05-19

**Authors:** Anne E. Taylor, Camille Ottoman, Frank Chaplen

**Affiliations:** ^1^Department of Crop and Soil Science, Oregon State University, Corvallis, OR, United States; ^2^Department of Biological and Ecological Engineering, Oregon State University, Corvallis, OR, United States

**Keywords:** thermodynamics, respiration, N mineralization, nitrification, inorganic soil N

## Abstract

Considerable research has shown that modifications in global temperature regimes can lead to changes in the interactions between soil respiration and the sequestration of C and N into soil organic matter (SOM). We hypothesized that despite the interconnected nature of respiration, net N mineralization, and nitrification processes, there would be differences in their thermodynamic responses that would affect the composition of inorganic soil N and the potential for retention of N in SOM. To test this hypothesis, soil respiration, N mineralization and nitrification responses were evaluated during constant temperature incubations at seven temperatures (4–42°C) in tilled and no-till soils from two major agroecological zones in Oregon; Willamette Valley, and Pendleton located in the Columbia River Basin. We observed (1) significant thermodynamic differences between the three processes in all soils, (2) a distinctly different thermodynamic profile in Willamette vs. Pendleton, and (3) a dynamic response of T_opt_ (optimal temperature for activity), and T_smax_ (temperature of greatest rate response to temperature), and temperature sensitivity (Δ${\text{C}}_{\text{p}}^{\text{‡}}$) over the incubation time course, resulting in shifts in the thermodynamic profiles that could not be adequately explained by changes in process rates. We found that differences in contributions of ammonia oxidizing archaea and bacteria to nitrification activity across temperature helped to explain the thermodynamic differences of this process between Willamette and Pendleton soils. A two-pool model of SOM utilization demonstrated that the dynamic thermodynamic response of respiration in the soils was due to shifts in utilization of labile and less-labile pools of C; and that the respiration response by Pendleton soils was more dependent upon contributions from the less-labile C pool resulting in higher T_opt_ and T_smax_ than Willamette soils. Interestingly, modeling of N mineralization using the two-pool model suggested that only the less-labile pool of SOM was contributing to N mineralization at most temperatures in all soils. The difference in labile and less-labile SOM pool utilization between respiration and N mineralization may suggest that these processes may not be as interconnected as previously thought.

## Introduction

**Figure 1:**
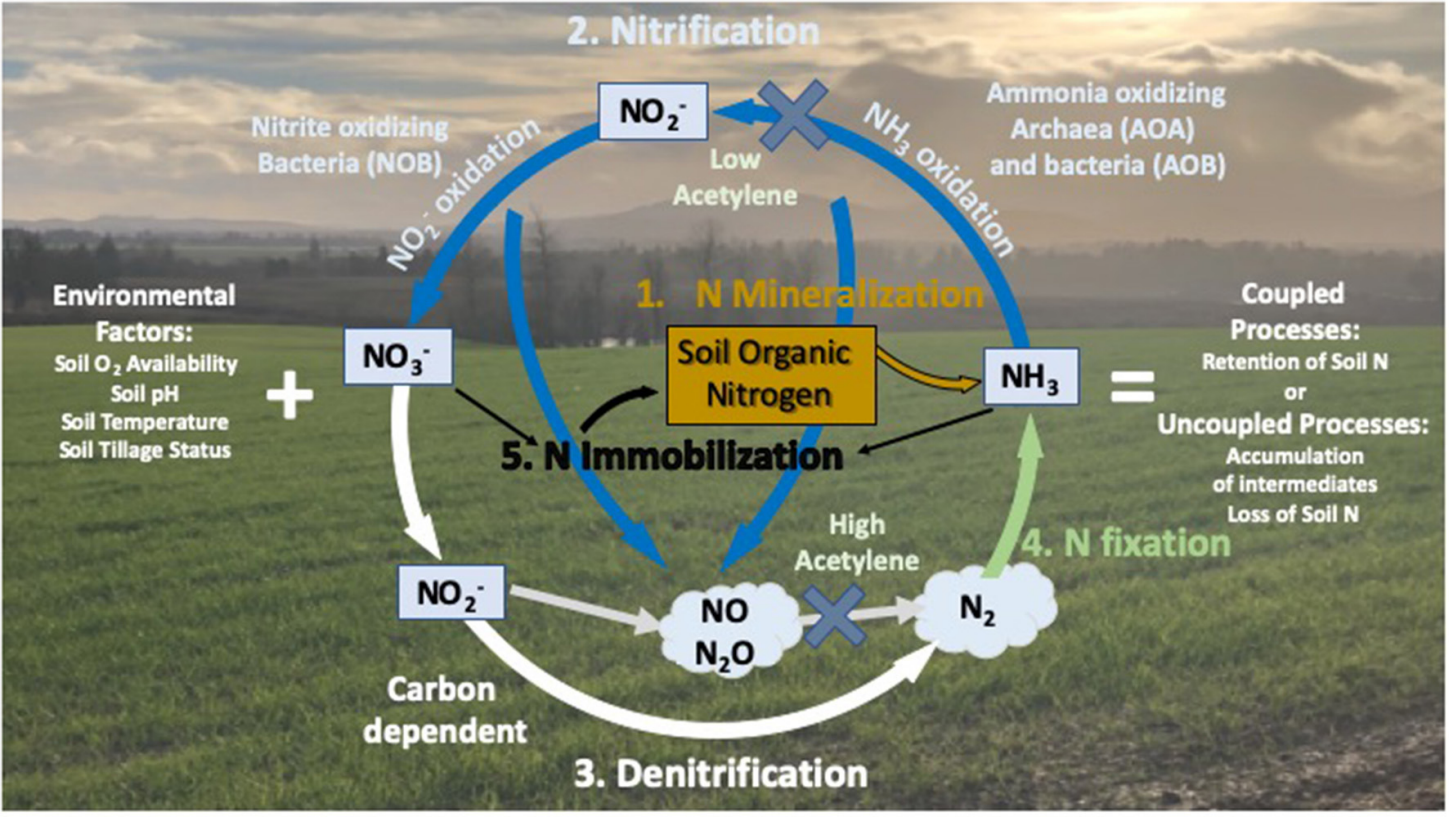
Nitrogen cycling in terrestrial systems. N cycling in terrestrial systems is microbially mediated with heterotrophic microorganisms mineralizing soil organic nitrogen (Step 1) to ammonium/ammonia (${\text{NH}}_{4}^{+}$/NH_3_), oxidation of NH_3_ to nitrite (${\text{NO}}_{2}^{-}$) then nitrate (${\text{NO}}_{3}^{-}$, Step 2) by nitrifying microorganisms, and denitrification to N oxide gases and dinitrogen gas (Step 3). Inorganic N is recovered through N fixation of dinitrogen through microbial action and natural events (lightning as an example) to form NH_3_ (Step 4), and inorganic N returns to the soil organic nitrogen pool through N immobilization by microorganisms (Step 5). Microbial N cycling in soils is a key regulator of N availability to plants and other players in terrestrial system. The addition of acetylene at varying levels affects different steps in the N cycle.

Considerable research has shown that modifications in global temperature regimes can lead to changes in the interactions between soil respiration and the sequestration of C and N into soil organic matter (SOM) (Davidson and Janssens, 2006; Sierra, 2012; Sierra et al., 2015; Blagodatskaya et al., 2016) (Figure 1). Warming of soils increases the rate of decomposition of SOM catalyzed by heterotrophic soil microorganisms for energy generation (CO_2_ respiration) and growth (C sequestration). A portion of the mineralized soil ${\text{NH}}_{4}^{+}$ released during respiratory processes is immobilized into microbial biomass, and the remaining ${\text{NH}}_{4}^{+}$ becomes available for plant uptake or nitrification and denitrification processes (De Neve et al., 2003; Miller and Geisseler, 2018). It is well-documented that the activity of SOM degrading microorganisms continues to increase in response to temperature up to ≥50°C, yet microbial growth is generally optimal at ~30°C (He et al., 2014; Dan et al., 2019); resulting in greater vulnerability of soil C and N loss above 30°C. Mineralization in the absence of microbial growth could lead to both reduced soil C storage, and production of mineral ${\text{NH}}_{4}^{+}$ in excess of plant demand. Under these conditions ${\text{NH}}_{4}^{+}$ becomes vulnerable to microbial oxidation to water-mobile ${\text{NO}}_{3}^{-}$ by soil nitrifying microorganisms which have demonstrated sustained activity at temperatures up to 42°C (Tourna et al., 2011; Taylor et al., 2017, 2019). The activity of nitrifiers controls the portion of N that is potentially vulnerable to leaching from the rooting zone in response to precipitation or irrigation, but also contributes to the efficiency with which mineral N is converted into plant N by controlling the availability of reduced (${\text{NH}}_{4}^{+}$) or oxidized (${\text{NO}}_{3}^{-}$) forms of N.

The assumption is generally made that soil microbes are C rather than N limited, and the release of mineralized ${\text{NH}}_{4}^{+}$ is the result of microbes mining the soil for C to fuel respiration (Hobbie and Hobbie, 2013; Farrell et al., 2014). The accumulation of net N is dependent upon both the rate of ${\text{NH}}_{4}^{+}$ released during mineralization, and the rate of immobilization of ${\text{NH}}_{4}^{+}$ into biomass and soil properties (Barrett and Burke, 2002; Pries et al., 2017). Nitrifier activity in turn is limited by the rate of N mineralization and competition with heterotrophic ${\text{NH}}_{4}^{+}$ immobilization. This study was initiated to determine if the trends observed in the temperature related thermodynamic responses of these three interrelated soil activities (respiration, net N mineralization, and nitrification) were similar, or if they differed in ways that affected mineral N production and speciation across temperatures. Previously we have found that the thermodynamic response of potential rates of nitrification in the absence of ${\text{NH}}_{4}^{+}$ limitations were similar across ecosystems (Taylor et al., 2017, 2019); but the thermodynamic response of nitrification in response to mineralized ${\text{NH}}_{4}^{+}$ may not follow the same pattern. Other researchers have found that the temperature response of N mineralization did not vary across regions (Miller and Geisseler, 2018), and that rates of soil respiration in non-desert biomes all had a similar temperature response (Carey et al., 2016). We hypothesized that despite the reliance of the rate of N mineralization upon respiration rates, and the reliance of the rate of nitrification upon N mineralization, there would be differences in the temperature response of these processes that would affect the composition of inorganic soil N and the possible retention of N in SOM.

To test this hypothesis, soil respiration, N mineralization and nitrification responses were evaluated during constant temperature incubations at seven temperatures (4–42°C) in soils from two major agroecological zones in Oregon with different temperature and rainfall regimes. To evaluate the effect that differences in SOM content exerted on C and N processes in response to temperature, we identified soils of the same soil type under conventional tillage (yearly cultivation) and conservative tillage (no or infrequent cultivation) management practices that have resulted in differences in soil C content. The temperature response of the rates of CO_2_ respiration, net N mineralization, and nitrification in each soil were modeled to obtain thermodynamic parameters to compare the processes. Additional modeling with multiple pool models were applied to gain a greater understanding of the dynamics in the thermodynamic response.

## Materials and Methods

Soils from two major Oregon agroecological zones were collected in June and July of 2019. Soils from OSU's Columbia Basin Agricultural Research Center (CBARC) in Pendleton Oregon are representative of the intermountain cereal cropping region of the Pacific Northwest that has a mean annual temperature (MAT) of 11.2°C (temperatures range −7.2–38.3°C), and receives ≤ 18 inches of precipitation annually. Pendleton soil is a Typic Haploxeroll (Walla Walla silt loam) in a winter wheat/fallow rotation in a long-term tillage fertility study since 1940. Three replicates of Till (annual tillage, pH 5.5–6.6) and No-till (established in 1982, pH 4.9–5.1) soils were sampled from the randomized block, split plot treatments receiving 80 lbs. N acre^−1^ during cropping years. Four to five soil samples were taken to a depth of 10 cm from each replicate plot via a random walk process. A composited sample was prepared for each replicate plot and brought to the laboratory.

The Willamette Valley sites have a MAT that is not significantly different from the Pendleton site (11.5°C); however, Willamette Valley soils rarely freeze and experience a lower maximum temperature (temperature range 3.3–33.9°C). The Willamette Valley receives an average of 42 inches precipitation yearly, and soils on the valley floor are often water saturated during winter and spring, and commonly used for crops tolerant to wet soil conditions such as spring grains, grass seed and pasture. Soils for this study were sampled from annual rye grass production fields belonging to a private grower under either Till (annual tillage and residue removal, pH 4.7–5.8) and No-till (infrequent tillage, mostly recently in 2016, 2013 and 2008; full residue load retained following harvest; pH 6.5–7.3) management, and receive 140 lbs. N acre^−1^ year^−1^. Both fields were on Dayton series silt loam soils (Vertic Albaqualfs) which are widely distributed in the Willamette Valley. The fields were large (70–80 acres each) and were sampled to yield three distinct field replicates. Soil microbial activity is greatest in surface soils, therefore four to five soil samples were taken to a depth of 10 cm from each replicate site via a random walk process, and a composited sample was prepared for each replicate site and brought to the laboratory.

In the laboratory all soils were sieved to <4.75 mm and stored at 4°C prior to experimentation. Total C and N were determined by standard methods of the Soil Health Lab at Oregon State University (Table 1). Ratios of C:N did not differ amongst the soils; however, WNT soils had total C and N contents significantly higher than the other soils, while values in PT soils were significantly lower (*p* ≤ 0.05).

**Table 1:** Selected properties of the soils utilized in this study.

**Soil**	**Total C** ** (%)**	**Total N** ** (%)**	**C:N**	**pH**
Willamette tilled	2.31 (0.28)b	0.19 (0.02)c	12.02 (0.33)a	5.15 (0.65)b
Willamette no-till	3.46 (0.30)c	0.29 (0.02)d	11.96 (0.53)a	6.89 (0.39)c
Pendleton tilled	1.13 (0.09)a	0.09 (0.01)a	12.30 (1.48)a	6.04 (0.55)b
Pendleton no-till	1.90 (0.17)b	0.15 (0.01)b	12.80 (0.47)a	4.96 (0.10)a

*Values indicate the average of three field replicates with standard deviation in parentheses. Lower case letters indicate significant differences in parameters (p ≤ 0.05)*.

### Mineralization Study

To evaluate the temperature response of soil respiration, N mineralization and nitrification, sampled soils were incubated moist, ~60% of field capacity, over a range of temperatures (4, 10, 16, 23, 30, 37, and 42°C) for 28 d and sampled weekly. Portions of moist soil (3 g) were weighed into glass tubes (one for each week) and placed within jars (500 ml volume) and sealed with a lid containing a septum. One set of samples was incubated in the presence of acetylene (10 μM in soil water) to evaluate the effect of temperature on ${\text{NO}}_{3}^{-}$ consumption, or acetylene-resistant heterotrophic nitrification, and another set of samples was incubated in the absence of acetylene to evaluate the response of autotrophic nitrification on inorganic N accumulation. At sampling times jars were opened and a tube of soil removed; the jar was left open for 10 min to allow for air exchange before being resealed. Acetylene treatments were reapplied and jars returned to temperature-controlled incubators. Soil portions removed from jars were decanted into 155 ml bottles with 15 ml dH_2_O and shaken ~250 rpm for 15 min at room temperature. Soil slurry aliquots (1.8 ml) were removed into microcentrifuge tubes, centrifuged for 3 min at 13.4 × 10^3^ g, and ${\text{NO}}_{2}^{-}$ + ${\text{NO}}_{3}^{-}$ concentrations were determined immediately on the supernatants as described previously and expressed on an oven-dried weight of soil basis (Giguere et al., 2015). Initial concentrations of ${\text{NO}}_{2}^{-}$ + ${\text{NO}}_{3}^{-}$ were subtracted from all time points. There was no significant change in background ${\text{NO}}_{2}^{-}$ + ${\text{NO}}_{3}^{-}$ concentrations in plus acetylene controls in any of the soils (data not shown), indicating there was no significant ${\text{NO}}_{2}^{-}$ + ${\text{NO}}_{3}^{-}$ sink, and no significant contribution to nitrification by heterotrophic organisms. The accumulation of ${\text{NO}}_{2}^{-}$ + ${\text{NO}}_{3}^{-}$ over time was considered to be the rate of soil nitrification. KCl was added to the soil slurry bottles (final concentration 2M) and the slurries shaken for 1 h. Soil slurry aliquots (1.8 ml) were removed into microcentrifuge tubes, centrifuged for 3 min at 13.4 × 10^3^ g, and extractable ${\text{NH}}_{4}^{+}$ concentrations were determined immediately on the supernatants as described previously and expressed on an oven-dried weight of soil basis (Giguere et al., 2015). Initial concentrations of ${\text{NH}}_{4}^{+}$ were subtracted from all time points. The accumulation of ${\text{NO}}_{2}^{-}$ + ${\text{NO}}_{3}^{-}$ and ${\text{NH}}_{4}^{+}$ over time was considered to be the rate of soil N mineralization.

To determine the soil respiration response to temperature, 10 g of moist soil were placed into 500 ml jar and sealed with a lid with a septum to allow for gas sampling, and incubated at the temperatures described above. Headspace samples in jars were evaluated for CO_2_ accumulation at each sampling time using a Picarro G2101-i Analyzer equipped with a multiport-valve sampler (Picarro Inc., Santa Clara, CA, USA). After CO_2_ measurement, jars were opened for 10 min to allow for air exchange before being resealed and CO_2_ again measured. CO_2_ that accumulated between one sampling and the next was expressed on an oven-dried weight of soil basis. The accumulation of CO_2_ over time was considered to be the rate of soil respiration. Total C and N were determined at the OSU Central Analytical Laboratory on pre- and post-incubation soil samples.

### Potential Assays

To evaluate the contributions of NH_3_-oxidizing archaea (AOA) and bacteria (AOB) to soil nitrification, the NH_3_ oxidation potential (AOP) response was determined on field soil samples as described previously (Taylor et al., 2017). Some AOP treatments included octyne (4 μM) to distinguish between AOA- and AOB-activities, using a procedure described by Taylor et al. (2013). AOA are octyne resistant, and AOB are octyne sensitive (total nitrification – nitrification by octyne-resistant AOA). AOP controls were comprised of soil suspensions to which acetylene was added (10 μM C_aq_) to evaluate the possibility of acetylene-resistant heterotrophic nitrification, and also to assess the significance of ${\text{NO}}_{3}^{-}$ consumption due to assimilation or denitrification. Each treatment was imposed on the individual soil replicates at each temperature. Samples were taken over the 48-h (16–42°C) or 72-h (4 and 10°C) incubations and accumulations of ${\text{NO}}_{2}^{-}+$${\text{NO}}_{3}^{-}$ were determined colorimetrically as previously described (Giguere et al., 2017). The accumulation of ${\text{NO}}_{2}^{-}+$${\text{NO}}_{3}^{-}$ was considered to be the rate of AOP. There was no significant ${\text{NO}}_{2}^{-}$ or ${\text{NO}}_{3}^{-}$ consumption or production in plus acetylene controls (data not shown), indicating all ${\text{NO}}_{2}^{-}+$${\text{NO}}_{3}^{-}$ accumulation was likely due to ${\text{NH}}_{4}^{+}$-dependent nitrification, and that alternate sinks for ${\text{NO}}_{2}^{-}$ or ${\text{NO}}_{3}^{-}$ were not significant under AOP conditions.

### Modeling of the Temperature Response of Soil Processes

The rates of respiration, inorganic N mineralization and NH_3_ oxidation were determined as described above at each temperature (4–42°C) and the thermodynamic constants obtained as described previously (Taylor et al., 2017). The temperature response data of each soil replicate was modeled using the macromolecular rate theory (MMRT) utilized by Schipper et al. (2014), ourselves and others to model the temperature response of a variety of microbial activities, including nitrification and respiration (Alster et al., 2016). The MMRT model is described by the equation

(1) $$\begin{array}{l} \ln\left(k\right)=\ln\left(\frac{k_{B}T}{h}\right)-\frac{\Delta H{\;}_{To}^{‡}+\;\Delta C_{P}^{‡}\left(T-T_{o}\right)}{RT}\\ \;+\frac{\Delta S{\;}_{To}^{‡}\;+\;\Delta C_{P}^{‡}\left(lnT-{lnT}_{o}\right)}{R} \end{array}$$

where *k* is the rate constant, *T*_*o*_ is the reference temperature at which the fitting process is initiated, *k*_*B*_ is Boltzmann's constant, *h* is Planck's constant, and *R* is the universal gas constant (8.314 J K^−1^ mol^−1^). The experimental data were fit to the MMRT model using SigmaPlot (Systat Software, San Jose, CA) to find three unknown parameters: (i) the change of enthalpy (ΔH_*To*_^‡^), (ii) the change of entropy (ΔS_*To*_^‡^), and (iii) the change in heat capacity that is a measure of temperature sensitivity (ΔC_*P*_^‡^) and describes the temperature dependence of the rate of activity and determines the degree of curvature of the ln(*k*) vs. T plot (Hobbs et al., 2013). The temperature for optimum activity *T*_*opt*_ and the temperature where the activity rate shows maximum sensitivity to changes in temperature (*T*_*s*_*max*_) are calculated using the fitted parameters from Equation (1).

(2) $$\begin{array}{r} T_{opt}=\;\frac{\Delta H{\;}_{To}^{‡}+\;\Delta C_{P}^{‡}T_{o}}{-\Delta C_{P}^{‡}-R} \end{array}$$

(3) $$\begin{array}{r} T_{s\text{\_}\max}\;\sim \;\frac{T_{opt}}{1+\;\sqrt{\frac{k}{\Delta C_{P}^{‡}}}\;} \end{array}$$

### Two-Pool Model Parameter Estimation

The two-pool model was utilized as a starting point for data analysis (Benbi and Richter, 2002; Alster et al., 2016). The two-pool conceptual model posits that the available carbon and nitrogen in the soil consists of a labile pool that can be quickly consumed and a less labile pool that is consumed with a much longer time constant. The mathematical form of the model is shown in Equations (4) and (5)

(4) $$\begin{array}{r} \text{Respiration Rate}\left(\text{t}\right)\;={\text{L}}_{0\text{c}}{\text{e}}^{-\text{kct}}+{\text{R}}_{\text{c}} \end{array}$$

(5) $$\begin{array}{r} \text{Mineralization Rate}\left(\text{t}\right)\;={\text{L}}_{0\text{N}}{\text{e}}^{-\text{kNt}}+{\text{R}}_{\text{N}} \end{array}$$

where L_oc_ and L_oN_ are the respective initial sizes of the labile carbon and nitrogen pools, k_C_ and k_N_ are the first order rate constants for CO_2_ production and N mineralization. R_c_ and R_N_ are the asymptotic respiration and nitrogen mineralization rates, respectively; and have units of μmol C or N gram soil^−1^ day^−1^.

Parameter estimation was done using the integrated form of the model with the respiration and nitrogen data generated as described herein. This is equivalent to the First order plus Linear (FLIN) equation from Gillis and Price (2011), which describes the cumulative carbon dioxide emission and nitrogen consumption in soils over time. The integrated forms of Equations (4) and (5) are shown in Equations (6) and (7), respectively; with units of μmol C or N gram soil^−1^. The variation associated with the data fits were reduced using this approach because it avoided the approximations associated with generating the rate of CO_2_ or mineral N accumulation curves from the original respiration and N mineralization data.

(6) $$\begin{array}{r} \text{Respiration}\left(\text{t}\right)=\left({\text{L}}_{0\text{c}}/{\text{k}}_{\text{c}}\right)\left(1-{\text{e}}^{-\text{kct}}\right)+{\text{R}}_{\text{c}}\text{t} \end{array}$$

(7) $$\begin{array}{r} \text{Mineralization}\left(\text{t}\right)=\left({\text{L}}_{0\text{N}}/{\text{k}}_{\text{N}}\right)\left(1-{\text{e}}^{-\text{kNt}}\right)+{\text{R}}_{\text{N}}\text{t} \end{array}$$

Parameter values were estimated using Matlab R2020b (Mathworks, Inc.) and the lsqcurve fit function with a Step Tolerance of 10^−1^. Order of magnitude initial guesses for parameter values were provided for determining parameters. Lower bounds for parameter values were set to 0 to preserve the physical meaning of determined parameters and upper bounds were set to infinity (unbounded). The resulting parameters were subjected to Analysis of Variance (ANOVA) to determine whether parameter differences observed were statistically significant. The data at 4°C for all soil types was not amenable to Matlab data analysis and lsqcurve fit with the pool model approach because of the relatively small changes observed experimentally.

### Statistical Analysis

Analysis of variance (ANOVA) analysis using the Holm-Sidak method in SigmaPlot (Systat Software, San Jose, CA) was used to evaluate pairwise comparisons among temperatures within each soil to determine if there were significant differences in CO_2_, or inorganic N accumulations in the 28 d incubations at each temperature (4–42°C). ANOVA analysis was also utilized to determine if there were differences between soils (Willamette and Pendleton) and management practices (Tilled or No-till) at each temperature. Repeated measures analysis of variance (RM ANOVA) analysis using the Holm-Sidak method in SigmaPlot was used to determine if there were significant changes in CO_2_, and inorganic N accumulations of the three field replicates over the course of the 28 d incubations at each temperature. To determine the significant differences in AOA or AOB AOP activities over the 4–42°C range, ANOVA tests were performed in SigmaPlot on the three field replicates of each soil. Significant differences of pairwise comparisons were obtained using two-tailed Student's test.

## Results

### Dynamic Response of Soil Processes

#### Respiration

**Figure 2:**
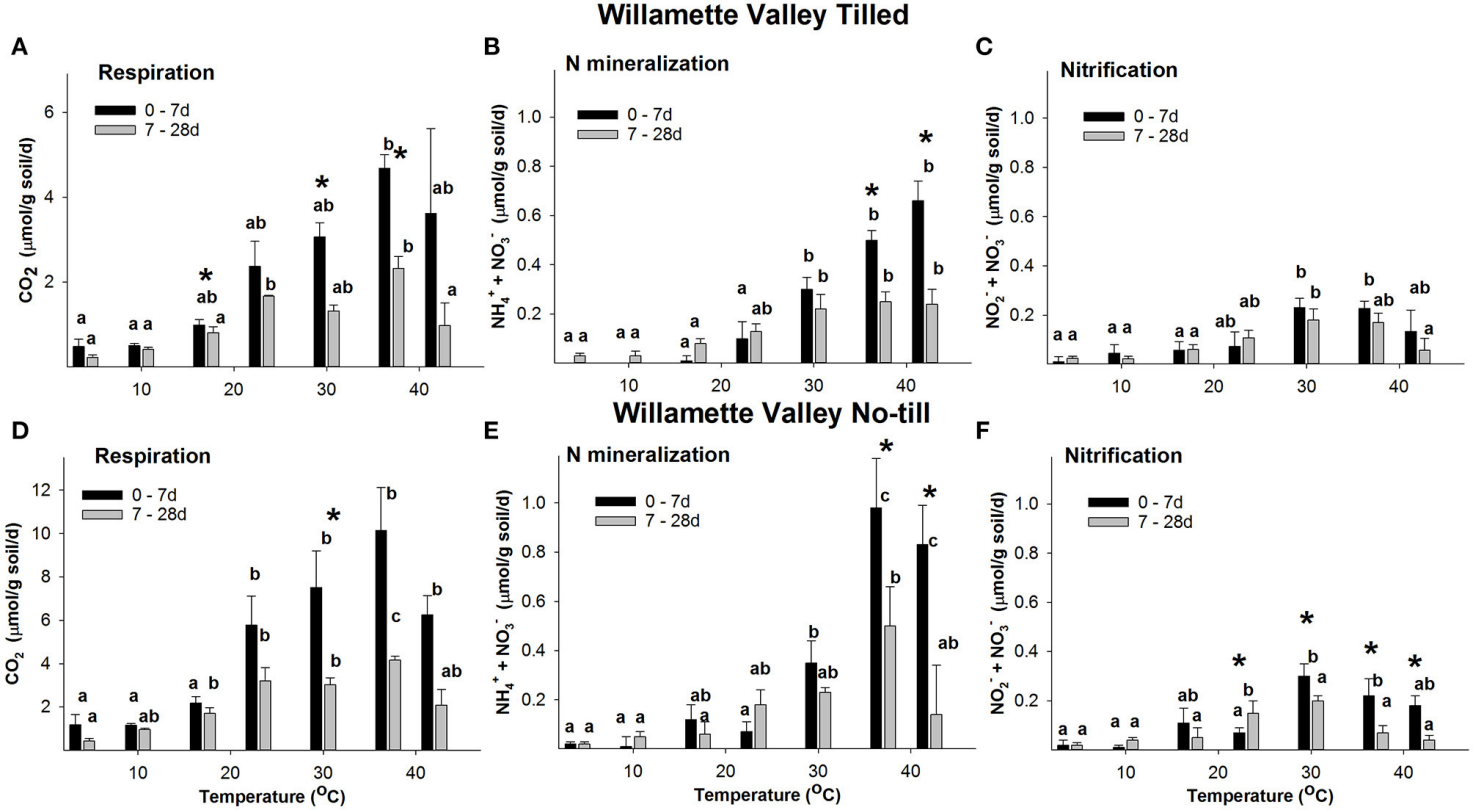
The rate response of respiration **(A,D)**, N mineralization **(B,E)**, and nitrification **(C,F)** in Willamette Valley soils across temperatures. Figure data are the average of three field replicates and the error bars represent the standard deviation. Lower case letters indicate where RM ANOVA analysis found significant differences in rates over the temperatures within a time period. Asterisks indicate where rates at 0–7 d differ significantly from 7 to 28 d (*p* ≤ 0.05).

**Figure 3:**
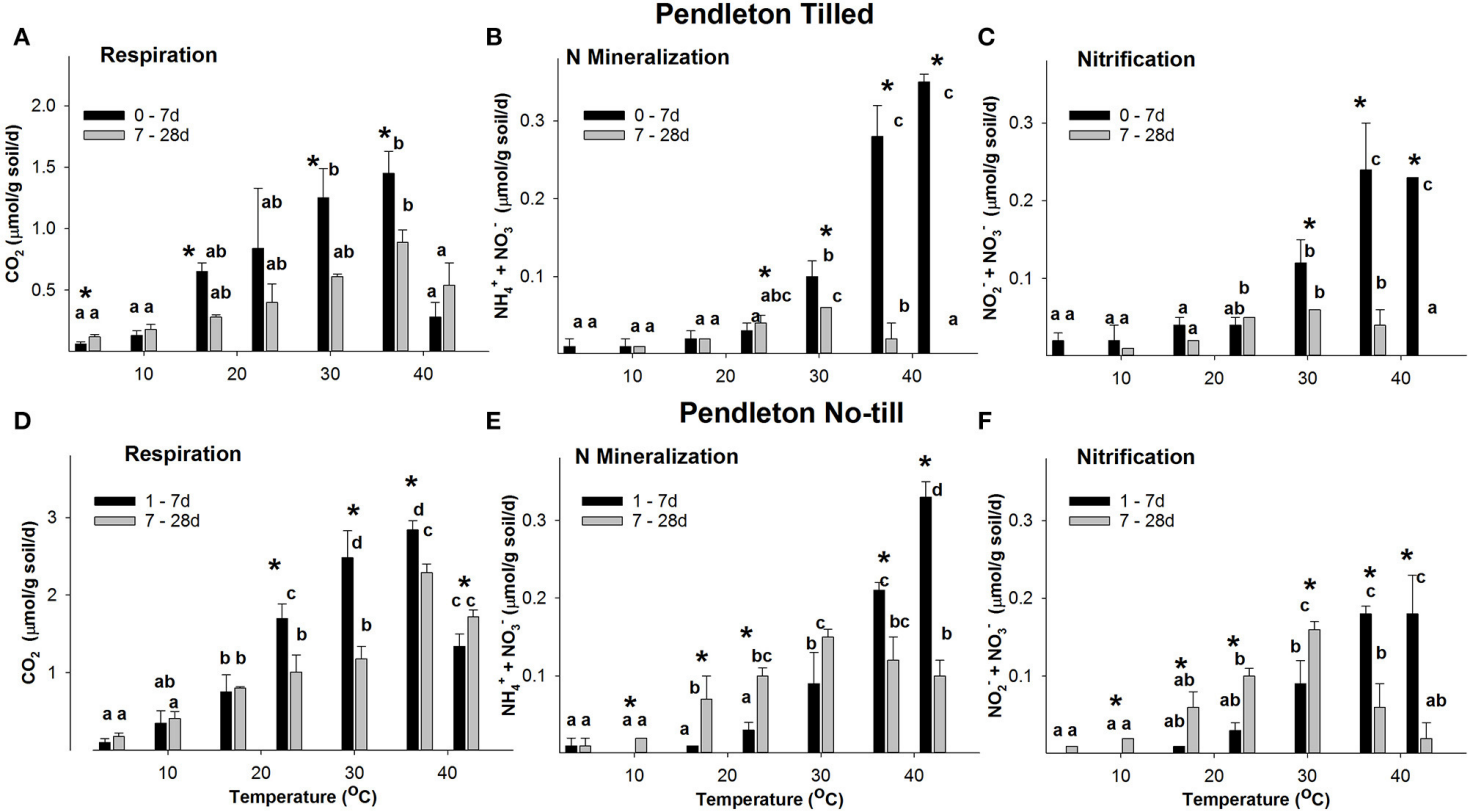
The rate response of respiration **(A,D)**, N mineralization **(B,E)**, and nitrification **(C,F)** in Pendleton soils across temperatures. Figure data are the average of three field replicates and the error bars represent the standard deviation. Lower case letters indicate where RM ANOVA analysis found significant differences in rates over the temperatures within a time period. Asterisks indicate where rates at 0–7 d differ significantly from 7 to 28 d (*p* ≤ 0.05).

Both Willamette and PNT soils maintained rates of CO_2_ production ~2-fold greater than their respective Tilled soils over the entire time course and at all temperatures. Respiration rates in Willamette soils at all temperatures were always ~2-fold greater than Pendleton soils of the same tillage treatment (Figures 2, 3). Repeated measures ANOVA indicated that there were significant increases in concentrations of CO_2_ in all soils and all temperatures over the entire time course (*p* ≤ 0.05, Supplementary Figure 1). However, beginning at 14 d rates of CO_2_ production slowed over time in all soils, declining significantly at some temperatures (Supplementary Figures 2, 3); with the exception of Pendleton soils incubated at 42°C in which respiration rates increased significantly after 7 d. Because initial respiration rates (0–7 d) differed from the secondary rates (7–28 d), further analysis compared these two time periods (Figures 2, 3). Highest rates of initial respiration were observed at 37°C in all four soils, as were the secondary rates of respiration in No-till soils and the PT soil. In WT soils, however, the temperature optimum of the secondary rates spanned 23, to 37°C.

#### N Mineralization

Willamette and PNT soils maintained rates of net ${\text{NH}}_{4}^{+}$ + ${\text{NO}}_{3}^{-}$ accumulation ~2-fold greater than their Tilled soil counterparts over the entire time course at temperatures ≥30°C. N mineralization rates in Willamette soils at temperatures ≥30°C were ~3-fold greater than Pendleton soils of the same management type (Figures 2, 3). Repeated measures ANOVA indicated there were significant accumulations of ${\text{NH}}_{4}^{+}$ + ${\text{NO}}_{3}^{-}$ over the 28 d incubations in all but a few instances of the four soils (*p* ≤ 0.05, Supplementary Figure 1). For example, in the 4°C incubations of both Tilled soils there was no significant difference between inorganic N levels at the beginning and end of the incubation, despite CO_2_ accumulating at this temperature in both soils over 28 d. Also, in the 37 and 42°C incubations of PT soils, ${\text{NH}}_{4}^{+}$ + ${\text{NO}}_{3}^{-}$ accumulated over the first 7 d of incubation and did not increase significantly afterwards.

A consistent feature of the temperature response of net N mineralization was that in all four soils the rate of N mineralization declined significantly over the 28 d incubation at 37 and 42°C (statistics shown in Supplementary Figures 2, 3). However, at temperatures ≤ 30°C, with only one exception (PT soil at 30°C), rates of ${\text{NO}}_{3}^{-}$ + ${\text{NH}}_{4}^{+}$ accumulation either did not significantly change over the course of the incubation, or increased significantly with time.

#### Nitrification

There were significant increases in ${\text{NO}}_{2}^{-}$ + ${\text{NO}}_{3}^{-}$ in all soils over the time course (*p* ≤ 0.05, Supplementary Figure 1). In acetylene treated controls there were no significant changes in background ${\text{NO}}_{2}^{-}$ + ${\text{NO}}_{3}^{-}$ concentrations in any of the soils (RM ANOVA *p* > 0.05), indicating that acetylene effectively inhibited nitrification and that there was no significant ${\text{NO}}_{3}^{-}$ sink (immobilization or denitrification) over the time course of the incubation (data not shown).

In WT soil there was no significant difference in the initial (0–7 d) and secondary (7–28 d) rates of nitrification at any temperature (Figure 2C). In contrast, in WNT soil the rate of nitrification declined significantly over 28 d at 30, 37, and 42°C; but, at temperatures ≤ 23°C rates of ${\text{NO}}_{3}^{-}$ accumulation either did not significantly change over the course of the incubation, or increased significantly with time (Figure 2F). In Willamette soils there was a significant difference in the rates of nitrification between Till and No-till soils only at 37°C, where Tilled soil had a ~2.2-fold greater rate than the No-till soil.

In both PT and No-till soils the rate of nitrification declined significantly over the 28 d incubation at 37 and 42°C; but in PT soil at temperatures ≤ 23°C rates of ${\text{NO}}_{2}^{-}$ + ${\text{NO}}_{3}^{-}$ accumulation did not change significantly over the course of the incubation, and in PNT soils rates of nitrification increased significantly with time at 10, 17, 23, and 30°C (Figures 3C,F). Rates of nitrification only differed between Pendleton Till and No-till soils over 0–7 d at 17°C (4.3-fold greater rates in Tilled soil), and over 7–28 d at 17 and 30°C (~2.7-fold higher in No-till).

### Thermodynamic Profiles

**Figure 4:**
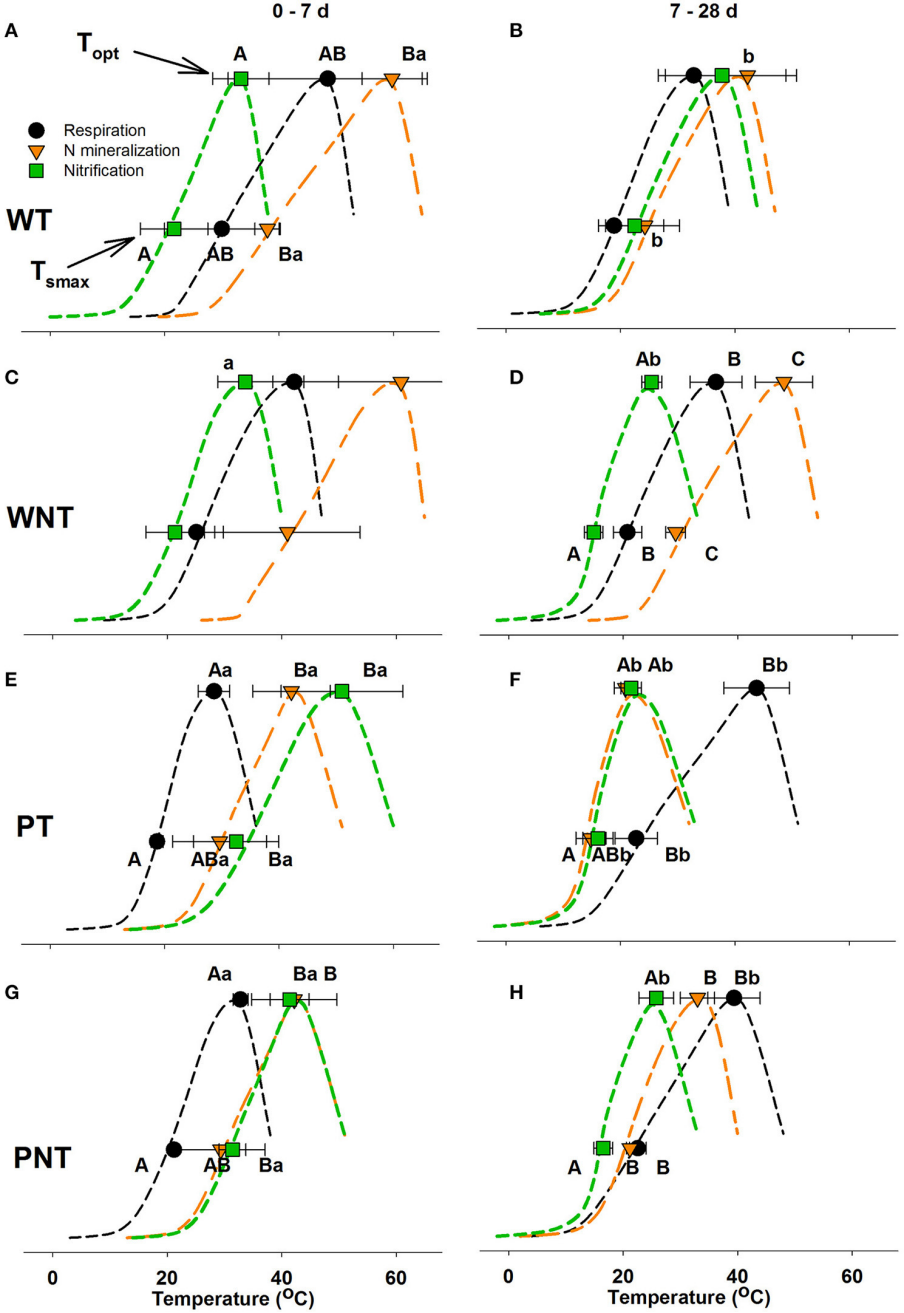
Comparison of maximum sensitivity to temperature change (T_smax_) and the optimal temperature (T_opt_) of respiration, net N mineralization, and net nitrification over 0–7 d **(A,C,E,G)** and 7–28 d **(B,D,F,H)**. The symbols represent the average value of the thermodynamic parameters determined for three field replicates of each of the four sampled soils, and the error bars represent the standard deviation of the average. The dashed lines are presented to aid the visualization of the temperature response with an inflection point showing T_smax_ and T_opt_. Upper case letters indicate significant differences in T_smax_ or T_opt_ between the three processes within a time interval; and lower-case letters indicate significant difference in T_smax_ or T_opt_ of an individual process between the two time periods (*p* ≤ 0.05).

One of the motivations for this study was to compare the thermodynamic response of respiration, N mineralization and nitrification to evaluate the interactions between these processes. The dynamic response of the rates of respiration, N mineralization and nitrification often coincided with changes in T_opt_ (optimal temperature for activity), and T_smax_ (temperature of greatest temperature increase), and temperature sensitivity (Δ${\text{C}}_{\text{p}}^{\text{‡}}$) over the time course in all four soils, sometimes changing the thermodynamic profiles (Figure 4; Table 2).

**Table 2:** Thermodynamic parameters describing respiration (Resp), N mineralization (N min), and nitrification (Nit).

**Soil**	**Process**	**T**_****smax****_ **(**^****°****^**C)**		**T**_*******opt*******_ **(**^****°****^**C)**		**ΔC**_*******p*******_^****‡****^ **(kJ mol**^****−1****^ **K**^****−1****^**)**	
		**(0–7 d)**	**(7–28 d)**	**(0–7 d)**	**(7–28 d)**	**(0–7 d)**	**(7–28 d)**
WT	Resp	30 (10) AB	19 (3)	49 (17) AB	33 (5)	−2971 (1967)	−3892 (1417)
	N min	38 (2) bB	24 (6) a 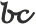	60 (5) bB	42 (9) a 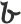	−1889 (907) 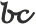	−2375 (616) 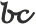
	Nit	22 (6) A	23 (5)	33 (5) A	38 (11)	−5816 (2292)	−5044 (5104) 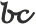
WNT	Resp	25 (5)	21 (3) B	42 (8)	36 (5) B	−2673 (792)AB	−3130 (947)B
	N min	41 (13)	29 (2) C 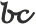	61 (17)	48 (5) C 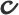	−2255 (885)B 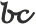	−2262 (874)B 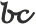
	Nit	21 (5)	15 (2) A	34 (5) a	25 (2) bA	−4821 (495)aA	−6722 (280)bA 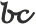
PT	Resp	19 (1) A	23 (4) B	29 (3) aA	44 (6) bB	−7599 (2365)a	−1681 (341)bA
	N min	30 (8) bAB	15 (3) aA 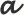	42 (7) bB	21 (2) aA 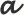	−5085 (1537)a 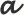	−19599 (5158)bB 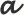
	Nit	33 (7) bB	16 (3) aAB	51 (11) bB	22 (2) aA	−2443 (845)a	−21570 (6076)bB 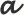
PNT	Resp	21 (1) A	23 (2) B	33 (1) aA	39 (5) bB	−5436 (741)a	−2732 (878)b
	N min	29 (8) AB	21 (1) AB 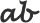	42 (7) B	33 (3) B 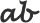	−4699 (1028) 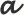	−6140 (3861) 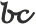
	Nit	31 (2) bB	17 (2) aA	41 (3) bB	26 (3) aA	−7993 (1994)	−8615 (2718) 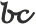

*Values are the average of three field replicates with standard deviation in parentheses. Lower case letters indicate a significant difference in parameters between time intervals, and upper case letters indicate a significant difference in parameter values between processes within an individual soil (p ≤ 0.05). Significant differences in the thermodynamic response across sampling sites are indicated by bold lower case script (p ≤ 0.05)*.

In both Willamette soils at 0–7 d, T_smax_ and T_opt_ of nitrification occurred at significantly lower temperatures than that of N mineralization, while the thermodynamic response of respiration was intermediate (Figure 4; Table 2). In WT soil T_smax_ and T_opt_ of N mineralization declined significantly at 7–28 d resulting in thermodynamic responses of respiration, N mineralization and nitrification that were not significantly different. However, in WNT soils the offset between the thermodynamic responses strengthened over time so that there were significant differences between the three processes at 7–28 d, with the temperature response of nitrification < respiration < N mineralization. In WT soil there were no changes in temperature sensitivity, Δ${\text{C}}_{\text{p}}^{\text{‡}}$, over the course of the incubation, and no significant differences in temperature sensitivity between the processes (Table 2). In contrast, in WNT soil temperature sensitivity of nitrification increased over time, and temperature sensitivity of nitrification was greater than that of N mineralization over the entire time course.

The Pendleton soils had distinctly different thermodynamic profiles than the Willamette soils (Figure 4). In both Pendleton soils over the 0–7 d period, T_smax_ and T_opt_ of N mineralization and nitrification were not significantly different, but were much higher than that of respiration. As the incubation progressed T_smax_ and T_opt_ of respiration in both Pendleton soils increased significantly at 7–28 d, while T_smax_ and T_opt_ of N mineralization and nitrification declined. In both Pendleton soils the temperature sensitivity of respiration declined over time (Table 2). However, in PT soil the sensitivity of N mineralization and nitrification increased significantly 7–28 d.

### Factors That Influence Differences Between Thermodynamic Profiles

Declines in values of T_smax_ and T_opt_ of nitrification over the time course in both Pendleton soils and in WNT soil may be explained by a greater contribution of AOB to nitrification activity as the incubation progressed. Previous studies have shown that soil AOB have lower T_smax_ and T_opt_ than AOA (Taylor et al., 2017; Duan et al., 2018). In nitrification potential assays of PT, PNT and WNT soil slurries, AOB nitrification activity dominated rates of potential nitrification ≤ 30°C (Supplementary Figure 4); whereas, in WT soil AOA made equal or greater contributions to nitrification activity at all temperatures. And in contrast to WT soil, in PT, PNT and WNT soils, rates of nitrification in response to N mineralization decreased significantly at 37 and 42°C (Figures 1, 2), temperatures at which AOA make up the largest share of potential activity. Together these data suggest that in PT and PNT soils that AOB increased contribution to the oxidation of mineralized ${\text{NH}}_{4}^{+}$ over the incubation leading to the significant declines in T_smax_ and T_opt_ of nitrification.

Changes in rates of N mineralization and respiration could not adequately explain shifts observed in the thermodynamic profiles of those processes. For example, although the significant decrease in T_opt_ of N mineralization of Tilled soils (Table 2) coincided with significant declines in rates of N mineralization at 37 and 42°C (Figures 2B, 3B); in No-till soils there were also significant declines in rates of N mineralization at 37 and 42°C (Figures 2E, 3E), yet there were no corresponding significant changes in T_opt_ of N mineralization in those soils. Thermodynamic parameters are properties of enzyme systems, not of substrates being utilized; however, we reasoned that a change in substrate utilization could over time influence the nature of the enzyme systems, actively turning over substrate, leading to changes in thermodynamic parameters. Therefore, in an effort to explain observed differences in thermodynamic parameters over time, a two-pool model of SOM utilization was utilized that discriminates between labile and less-labile pools of SOM (Supplementary Table 1). The analysis of CO_2_ accumulation over the incubations revealed that the size of the initial labile substrate pool (L_0_) increased significantly (*p* ≤ 0.05) with temperature in both Willamette soils, peaking at 37 or 42°C with 10.1–14.1 μmol C g soil^−1^; whereas PT soil had the largest L_0_ at 37°C (0.2 ± 0.2 μmol C g soil^−1^), and PNT at 23–30°C (9.9–10.6 μmol C g soil^−1^). There were also significant differences across temperature in the rate of utilization from the less-labile (R) SOM pool in all but WNT soil; with R contributing most to CO_2_ accumulation at 37°C in WT and PNT soil, and 30°C in PT soil.

**Figure 5:**
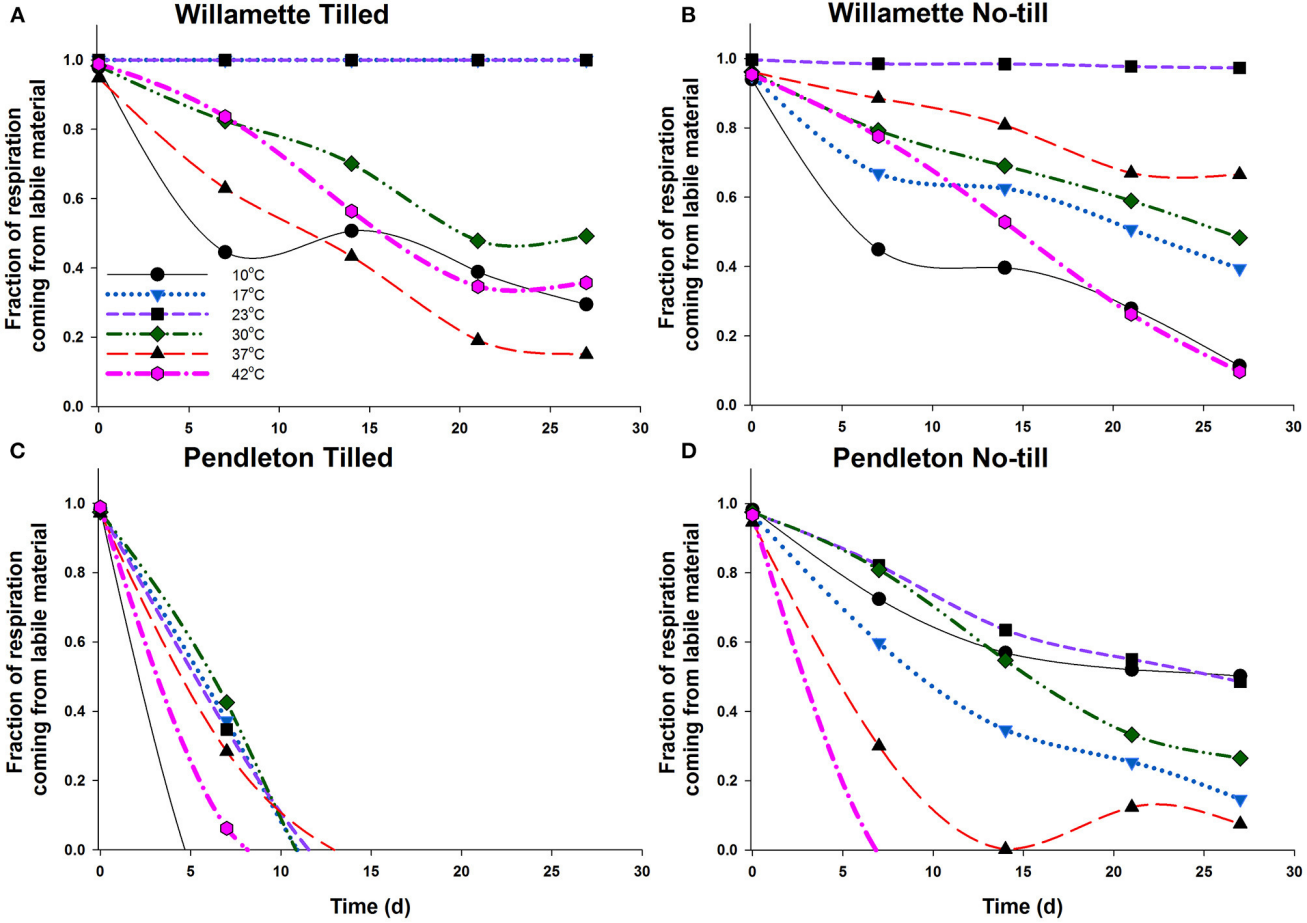
Fraction of the respired CO_2_ contributed by the labile SOM pool in Willamette Tilled **(A)**, Willamette No-till **(B)**, Pendleton Tilled **(C)**, and Pendleton No-till **(D)** soils. The two pool conceptual model estimates of the amount of CO_2_ contributed by the labile SOM pool as the soil incubation progressed. Lines represent the average of three soil replicates. Error bars were omitted to aid readability.

Using the two-pool model results, the fraction of labile SOC pool contributions to CO_2_ production over the course of the incubations were estimated (Figure 5). Model predictions did not differ significantly than the experimental data at most temperatures in all soils (Supplementary Figure 5; Supplementary Tables 3, 5), and Supplementary Figure 6 provides additional support for the two-pool assumption because the data approaches an asymptotic value (R_c_) within the timeframe of the experiment. Initially in both Willamette soils the labile pool of SOM at 10°C contributed less to respiration than at any other temperature. This could indicate that the labile pool at 10°C was too small to contribute much to respiration activity, or that the less-labile pool was readily available at 10°C and used to a greater extent. At all temperatures the labile SOM pool of the Willamette soils contributed to CO_2_ production over the entire 28 d incubations; however, as expected, the share of CO_2_ production from the labile pool decreased over time at most temperatures. The exceptions were WT soil at 23°C and WNT soil at 17 and 23°C where the labile SOM contributed nearly all of the respiration for the entirety of the incubation. Because the Willamette soils had sustained contributions of the labile SOM pool to CO_2_ production, there were no significant changes observed in thermodynamic parameters of respiration across the 28 d incubation (Figure 4; Table 2).

In PT soil the labile SOM pool contributed to CO_2_ production for <14 d at any temperature (Figure 5). Likewise, the labile SOM pool of PNT soil contributed to CO_2_ production for <14 d at 42 and 37°C, and at other temperatures the share of CO_2_ production from the labile pool decreased over time. The greater utilization of the less-labile R pool in Pendleton soils may have required enzyme systems that function at higher temperatures. And indeed, we found that these changes in substrate pool utilization in both Pendleton soils coincided with significant increases in T_smax_ and T_opt_ of respiration, and significant declines in temperature sensitivity (Table 2; Figure 4).

The two-pool model of the ${\text{NO}}_{3}^{-}$ + ${\text{NH}}_{4}^{+}$ accumulation data differed significantly from the two-pool model of CO_2_ accumulation (Supplementary Table 2). The extremely small size of the labile pool, coupled with its lack of response to temperature, and lack of significant difference in labile N availability between the four soils suggested that the labile pool did not contribute to biologically-mediated N mineralization. In contrast, the rate at which the less-labile pool of SOM was contributing to N mineralization did respond to temperature, and utilization of soil N from this pool peaked at 37 or 42°C in all soils. This could suggest that mineralized N comes from a different pool, or at least behaves differently than the C pool(s) contributing to respiration. While there were significant differences in the R pool size with temperature in each soil, and also significant differences between soils, this analysis did not yield insights into what caused the significant decreases in T_smax_ and T_opt_ of N mineralization in Willamette and PT soil. Because the two-pool model only showed that the R pool was contributing to inorganic N accumulation, it might be expected that there were more significant differences in the model and experimental data for N (Supplementary Tables 4, 6); and most of those were concentrated in PT soil which had the lowest soil N contents.

## Discussion

### Response of Respiration to Temperature

Regardless of differences in climatic regimes, or soil C contents, there were no significant differences in the thermodynamic parameters describing respiration of the four soils used in this study. This result might make intuitive sense because of the wide range, distribution, and niche redundancy of heterotrophic microorganisms that respire. Indeed, in an analysis of 27 temperature manipulation experiments, the respiration rates of all non-desert biomes followed the same Gaussian response (Carey et al., 2016). In addition, Alster et al. (2016) observed that microbial makeup only explained 30% of the variability in soil respiration response between soils, while soil type and size of soil C pool described the rest. In this study the T_opt_ determined using the MMRT model ranged from 28 to 49°C (Table 2), and were somewhat higher than the MMRT T_opt_ of 20–37°C of the labile C pool utilization determined in soils collected from sites on the Great Plains and incubated at a more limited number of temperatures (10, 20, and 30°C) (Alster et al., 2016). While rates of respiration were dynamic for all soils over the course of the incubation, in Willamette soils T_smax_, T_opt_ and temperature sensitivity of respiration (Δ${\text{C}}_{\text{p}}^{\text{‡}}$) did not change; however, in Pendleton soils T_opt_ increased significantly and there were significant increases in temperature sensitivity. This may indicate that there was a shift in substrate utilization in Pendleton soil from a labile to a less available C pool requiring a higher activation temperature. The decomposition response of organic matter in soil to temperature is considered to be an ecosystem property involving chemical, physical and biological factors and processes (Schmidt et al., 2011) with both “slow and fast” pools of soil organic matter contributing to soil respiration (Conant et al., 2011), and that the different pools have different properties of degradation (Capek et al., 2019). For instance, labile SOM utilization responds less to temperature increase than less-labile pools (Conant et al., 2011; Wang et al., 2020). A shift in substrate utilization in this study was confirmed by modeling CO_2_ accumulation using a two-pool model (Alster et al., 2016) which demonstrated that the initially available, or labile, substrate pool of Willamette soils did not become limiting over the course of the incubation; but that the PT soil labile C pool did not contribute to CO_2_ accumulation at any temperature after about 14 days, and that in PNT soils contributions to CO_2_ by the labile pool fell below 50% by the end of the incubation at 30–42°C (Figure 5).

### Response of N Mineralization to Temperature

The average T_opt_ of N mineralization in this study determined by MMRT was 43.6 ± 13.2°C. To our knowledge this is the first N mineralization study to be modeled by MMRT, but this value compares well to T_opt_ of 50°C determined by Beck (1983) and 37°C by DeNeve et al. (1996). In all four soils the rate of mineralization declined significantly over the 28 d incubation at 37 and 42°C, and was accompanied by a decrease in T_opt_ of N mineralization over time that was significant in Tilled soils. It has been suggested that the rate of dissolved organic matter production is the rate-limiting step in soil C and N mineralization (Bengtson and Bengtsson, 2007), and that the enzymatic depolymerization of SOM is the rate-limiting step in N mineralization (Schimel and Bennett, 2004). Temperature affects both production and turnover of extracellular enzymes in soils. Perhaps these changes in the thermodynamic properties of N mineralization indicate that sustained exposure to the higher temperatures had negative effects on the integrity of soil heterotrophic microorganisms, or lead to the denaturing of their extracellular enzymes, leading to a shift in substrate pool utilization at temperatures ≥37°C. In contrast, at temperatures ≤ 30°C, with only one exception (PT soil at 30°C), rates of ${\text{NO}}_{3}^{-}$ + ${\text{NH}}_{4}^{+}$ accumulation either did not significantly change over the course of the incubation, or increased significantly with time. An increase in the rate of inorganic N accumulation over time might indicate that biosynthetic activity was most active initially, and then as the incubation progressed, and N immobilization decreased, that inorganic N then accumulated in the incubations.

In a previous study conducted at 5, 15, and 25°C, the response of N mineralization to temperature did not vary across regions (Miller and Geisseler, 2018); however, we observed significant differences in T_smax_ and T_opt_ and Δ${\text{C}}_{\text{p}}^{\text{‡}}$ between Willamette and Pendleton soils in response to temperatures ranging from 4 to 42°C. While there were significant site differences (Willamette vs. Pendleton) in N mineralization thermodynamic parameters, differences in soil C contents of the Tilled and No-till soils of each site did not lead to significant differences in the properties of N mineralization within either site. This result is supported by previous comparisons of disturbed soils (lower total C) and undisturbed soils (higher total C) where the net N mineralization response did not differ significantly between the soils (Wang et al., 2006; Keiser et al., 2016). It was hypothesized that in soils with higher total C that heterotrophic microorganisms were immobilizing ${\text{NH}}_{4}^{+}$, reducing the apparent net ${\text{NH}}_{4}^{+}$ accumulation; and an evaluation of a large number of isotope pool dilution experiments revealed there was a strong positive correlation between the concentration of soil C, and both gross N mineralization and microbial assimilation of ${\text{NH}}_{4}^{+}$ (Booth et al., 2005).

There were significant differences between respiration and N mineralization thermodynamic parameters in WNT, PT and PNT soils (Table 2; Figure 4). PT soil, which had the lowest total C and N of the four soils in this study, had the greatest number of differences between T_smax_ and T_opt_ of respiration and N mineralization, and was the only soil in the study where temperature sensitivity was significantly different between these processes. It was previously observed that soil C decomposition and N mineralization have different responses to temperature (Shi et al., 2020), and that C and N cycling can be decoupled if microbes preferentially utilize nitrogen-containing components during the decomposition of soil organic matter (Knowles et al., 2010). In addition, the two-pool modeling of C and N utilization (Supplementary Tables 1, 2) indicated that the less-labile N pool was preferentially utilized while the labile C pool was being consumed. This agrees with the observation that labile C triggered a catabolic shift toward mining of the N-rich fraction of soil organic matter (Rousk et al., 2016).

### Response of Nitrification to Temperature

With the exception of PT soils where temperature sensitivity was significantly greater than the other soils by the end of the incubation, there were no significant differences in the thermodynamic parameters of nitrification between soils. This agrees with a previous study from our group that found the thermodynamic response of archaeal and bacteria ${\text{NH}}_{4}^{+}$-oxidizers were consistent across sites (Taylor et al., 2017), but contrasts with the finding of Duan et al. (2018) where soil types had a significant effect on AOA T_opt_, and the study by Gubry-Rangin et al. (2017) which found soil pH influenced the optimal temperature for AOA population increases. In this study we did not use selective inhibitors to discriminate between AOA and AOB activity, therefore the thermodynamic parameters represent the integration of both groups of soil nitrifiers. With the exception of WT, thermodynamic parameters of nitrification were dynamic over time and there were significant decreases in T_opt_, and T_smax_ over the course of the incubations, and in WNT temperature sensitivity increased significantly. These trends may indicate a shift from AOA to AOB dominated activity. Initially (0–7 d), the T_opt_ of nitrification in this study (40 ± 8°C) was more similar to those previously reported for AOA (33 ± 4°C and 34 ± 4°C)(Taylor et al., 2017; Duan et al., 2018), while over 7–28 d the T_opt_ of nitrification in this study (28 ± 7°C) suggested a larger contribution to soil nitrification activity by AOB (20 ± 3°C and 29 ± 1°C) (Taylor et al., 2017; Duan et al., 2018).

We could find just a handful of studies that have evaluated both net N mineralization and nitrification (Wang et al., 2006; Lauren et al., 2019; Mukai et al., 2020). Mukai et al. (2020) observed negative rates of ${\text{NH}}_{4}^{+}$ mineralization in the presence of nitrification, and Wang et al. (2006) found no significant difference between N mineralization and nitrification rates; however, in the Lauren et al. (2019) study accumulations of ${\text{NO}}_{3}^{-}$ were minimal and the contributions by nitrification to N mineralization could be ignored. In this study, we observed significant differences in the thermodynamic characteristics of nitrification and N mineralization in the Willamette soils. Early in the incubations (0–7 d) of WT soils and later in the WNT soils (7–28 d) T_smax_ and T_opt_ of N mineralization were significantly higher than those of nitrification which may suggest some uncoupling between nitrification and N mineralization. Since soil heterotrophs collectively have the ability to turn over SOM, it might be expected that there would be organisms that could respond at the high end of the temperature range. Nitrifiers are more phylogenetically constrained than heterotrophs, and we might expect the reduced phylogenetic diversity could lead to niches where nitrification falters compared to heterotrophic mineralization activity.

Despite dependence of nitrification on net mineralization (Keiser et al., 2016), soil N dynamics and nitrification are linked to C cycling (respiration) due to the competition of NH_3_-oxidizers and heterotrophs for mineralized ${\text{NH}}_{4}^{+}$; especially when the combination of temperature and substrate supply is favorable for microbial growth (Verhagen and Laanbroek, 1991; Booth et al., 2005). Significant differences were observed in this study between nitrification and respiration thermodynamic parameters in all but WT soils. In Pendleton soils T_smax_ and T_opt_ of nitrification were greater than respiration, but by the end of the incubation they were significantly lower; due to both an increase in T_opt_ of respiration in response to a shift to less-labile substrate utilization and a decrease in T_smax_ and T_opt_ of nitrification as NH_3_-oxidizers with thermodynamic parameters more like AOB contributed more NH_3_ oxidation activity. This may suggest that in this soil AOB are able to contribute more to nitrification when competition with heterotrophs for ${\text{NH}}_{4}^{+}$ had decreased once labile C resources were exhausted, or that over time a subpopulation of AOB with higher substrate affinity become more active. A similar decline in the T_opt_ of nitrification was also observed in WNT, although in this soil labile C contributed to respiration for the entire incubation and the thermodynamic parameters of respiration did not change. Previously Hoyle et al. (2006) found that soil nitrification was the principle process controlling ${\text{NH}}_{4}^{+}$ consumption in dry-land soils incubated above 20°C. These observations may support the idea that a group of nitrifiers with T_opt_ more like AOB were able to compete with heterotrophic organisms for ${\text{NH}}_{4}^{+}$ even when labile C was still abundant and conditions for growth were favorable.

### Where to Go From Here?

It has been observed previously that the characteristic thermodynamic response of soil nitrification, and N mineralization cannot be adequately described because of the interdependence of these processes on heterotrophic immobilization of N and C (Schutt et al., 2014). However, in this study we observed significant thermodynamic differences between the three processes in all our four soils, that could be explained by decoupling of C and N utilization by soil microbes, and a decoupling of N mineralization and nitrification. The extent of these differences lead to a distinctly different thermodynamic profile for the three process in Willamette vs. Pendleton soils and emphasizes the need to explore the relationship between these three processes in other soils. We also observed examples of a dynamic thermodynamic response of respiration, N mineralization and nitrification over the course of a 28 d incubation in one or more soils. This emphasizes the need for field or site-specific estimates of basic soil processes (respiration, N mineralization and nitrification) in order to support crop yields but also reduce surplus N addition to soils (Heumann et al., 2011).

## Data Availability Statement

The raw data supporting the conclusions of this article will be made available by the authors, without undue reservation.

## Author Contributions

AT was responsible for experimental design, data analysis, and writing of the manuscript. CO was responsible for incubations and data collection. FC was responsible for modeling, writing, and manuscript preparation. All authors contributed to the article and approved the submitted version.

## Conflict of Interest

The authors declare that the research was conducted in the absence of any commercial or financial relationships that could be construed as a potential conflict of interest.
