# Supplementary material for: Implications of the Thermodynamic Response of Soil Mineralization, Respiration, and Nitrification on Soil Organic Matter Retention
Source: Front Microbiol. 2021 May 19;12:651210. doi: 10.3389/fmicb.2021.651210 (PMC8170049; doi:10.3389/fmicb.2021.651210)
Supplement: Supplementary file 1 [file Data_Sheet_1.pdf]

Supplemental Table 1. Parameters determined utilizing a two-pool method to model the CO_2 accumulation over the course of the 28d incubations to discriminate between the utilization of labile and recalcitrant pools of SOM. Values represent the average value (± standard deviation) of initial labile pool size (L_0), first-order rate of labile pool utilization (k), and the rate of recalcitrant SOM utilization (R) at each temperature. Lower case letters indicate a significant difference in parameters within a soil between temperatures, and upper case indicate a significant difference between soils at a given temperature ($0 \leq 0.05$).

Respiration

Soil	Temperature (°C)	L_0 (μmol C g soil^{-1})	k (d^{-1})	R (μmol C g soil^{-1} d^{-1})
WT	10	0.16 (0.27) a	1.08 (1.86)	0.26 (0.23) ab A
	17	1.16 (0.12) a B	0.02 (0.01)	0.00 (0.00) a
	23	2.53 (0.39) ab B	0.02 (0.01)	0.00 (0.00) a A
	30	4.68 (0.21) ab	0.13 (0.02)	0.81 (0.09) b
	37	6.89 (0.81) b C	0.12 (0.02)	1.94 (0.37) c
	42	10.12 (4.41) c B	0.21 (0.18)	0.49 (0.28) ab A
WNT	10	0.55 (0.61) a	0.01 (0.01)	0.69 (0.20) B
	17	2.51 (1.23) ab B	0.06 (0.02)	0.82 (0.48)
	23	6.22 (1.67) ab C	0.04 (0.01)	0.07 (0.11) A
	30	7.03 (2.06) ab	0.15 (0.10)	2.09 (0.87)
	37	14.37 (7.48) b BC	0.07 (0.06)	1.42 (1.88)
	42	14.14 (6.36) b B	0.12 (0.07)	1.28 (1.21) B
PT	10	0.00 (0.00) a	4.92 (5.30)	0.17 (0.04) a A
	17	0.02 (0.00) ab A	0.22 (0.03)	0.41 (0.04) ab
	23	0.06 (0.05) ab A	0.16 (0.05)	0.49 (0.21) b B
	30	0.08 (0.04) ab	0.14 (0.06)	0.73 (0.07) c
	37	0.23 (0.17) b A	0.10 (0.04)	1.04 (0.12) d
	42	0.03 (0.01) ab A	0.18 (0.21)	0.46 (0.09) abc A
PNT	10	0.67 (0.32) a	0.05 (0.01) ab	0.18 (0.05) a A
	17	0.89 (1.04) a A	0.07 (0.05) ab	0.56 (0.38) ab
	23	10.63 (1.25) b D	0.19 (0.00) b	0.60 (0.15) ab B
	30	9.87 (6.34) b	0.15 (0.11) ab	0.80 (0.10) ab
	37	2.35 (1.26) ab B	0.03 (0.02) ab	1.32 (1.20) b
	42	0.27 (0.42) a A	0.01 (0.01) a	1.37 (0.37) ab AB

Supplemental Table 2. Parameters determined utilizing a two-pool method to model the NO_3^- + NH_4^+ accumulation over the course of the 28d incubations to discriminate between the utilization of labile and recalcitrant pools of SOM. Values represent the average value (± standard deviation) of initial labile pool size (L_0), first-order rate of labile pool utilization (k), and the rate of recalcitrant SOM utilization (R) at each temperature. Lower case letters indicate a significant difference in parameters within a soil between temperatures, and upper case indicate a significant difference between soils at a given temperature (0 ≤ 0.05).

N mineralization

Soil	Temperature (°C)	L_0 (µmol N g soil^{-1})	k (d^{-1})	R (µmol N g soil^{-1} d^{-1})
WT	10	0.013 (0.022)	8.36 (4.10) ab	0.04 (0.02) a AB
	17	0.001 (0.000)	3.44 (5.95) ab	0.07 (0.01) a B
	23	0.002 (0.001)	7.39 (6.12) b	0.10 (0.04) a AB
	30	0.005 (0.002)	0.18 (0.08) a	0.22 (0.05) b C
	37	0.015 (0.012)	0.18 (0.06) a	0.29 (0.02) bc AB
	42	0.007 (0.006)	0.08 (0.14) a	0.34 (0.04) c B
WNT	10	0.000 (0.000)	0.000 (0.000)	0.04 (0.00) a B
	17	0.001 (0.001)	0.670 (0.596)	0.05 (0.04) a B
	23	0.002 (0.001)	0.001 (0.002)	0.13 (0.05) a B
	30	0.450 (0.397)	0.155 (0.092)	0.16 (0.01) ab BC
	37	1.10 (1.16)	0.466 (0.664)	0.45 (0.21) b B
	42	1.60 (0.30)	0.251 (0.276)	0.08 (0.14) a A
PT	10	0.000 (0.000)	5.53 (4.80) c	0.02 (0.00) a AB
	17	0.000 (0.000)	4.33 (2.77) b	0.03 (0.01) ab A
	23	0.000 (0.000)	0.001 (0.001) a	0.04 (0.01) b A
	30	0.001 (0.000)	0.16 (0.25) a	0.07 (0.01) c A
	37	0.001 (0.000)	0.69 (0.60) a	0.12 (0.01) d A
	42	0.002 (0.001)	0.82 (0.71) a	0.12 (0.02) d A
PNT	10	0.000 (0.000)	2.38 (4.13)	0.01 (0.00) a A
	17	0.000 (0.000)	3.75 (6.50)	0.06 (0.02) b AB
	23	0.000 (0.000)	0.00 (0.00)	0.08 (0.01) b AB
	30	0.001 (0.001)	0.00 (0.00)	0.14 (0.02) c B
	37	0.003 (0.000)	0.34 (0.30)	0.16 (0.01) c AB
	42	0.003 (0.000)	0.53 (0.30)	0.17 (0.01) c AB

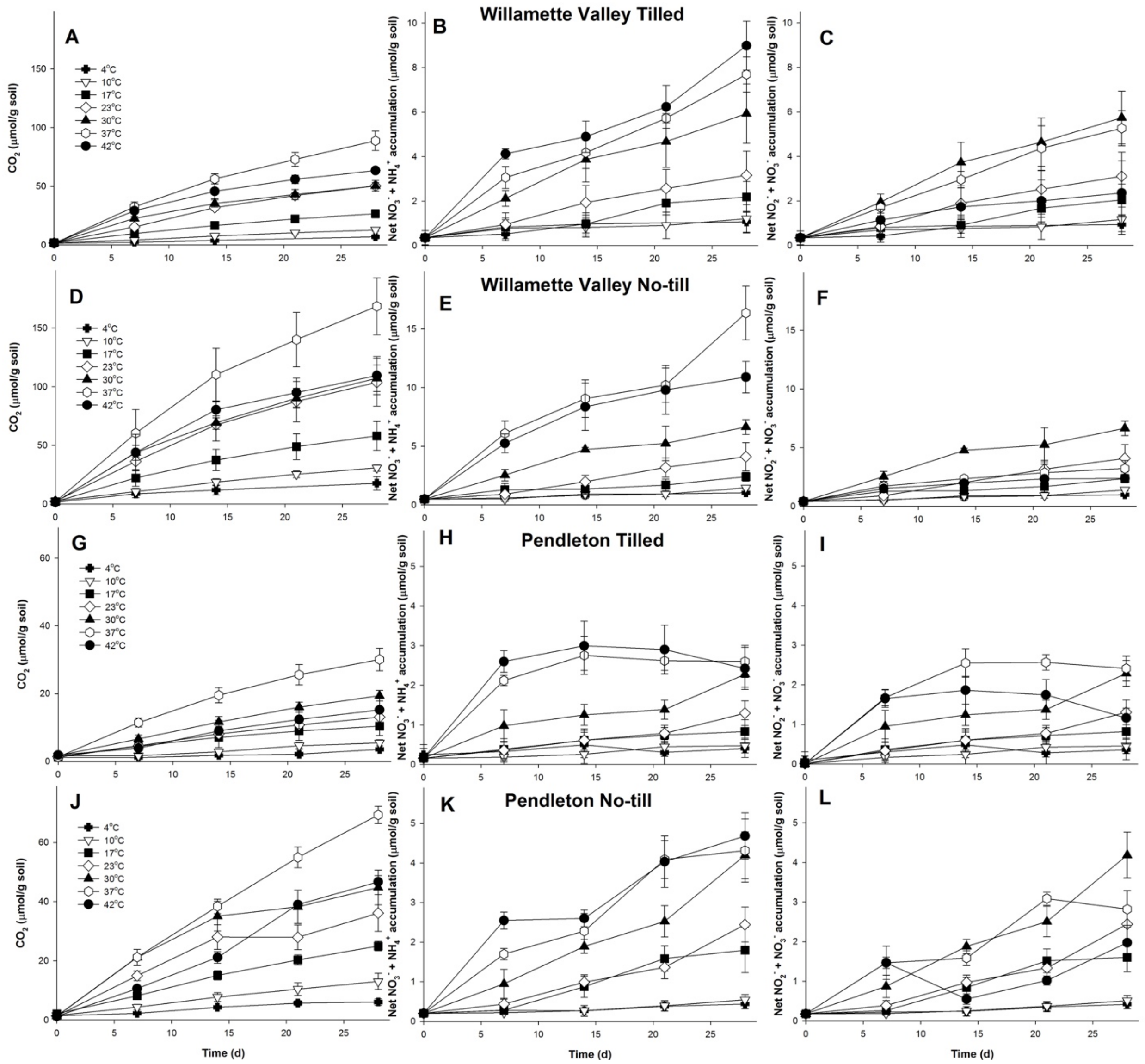

Supplemental Figure 1. The accumulation of CO_2 and inorganic N (NH_4^+ and NO_3^-) in Willamette and Pendleton soils incubated over a range of temperatures for 28d. Sealed jars were incubated at indicated temperatures. Error bars represent the standard deviation of the average of three field replicates. RM ANOVA indicates that there were significant increases in CO_2 in all soils and all temperatures over the time course ($p \leq 0.05$).

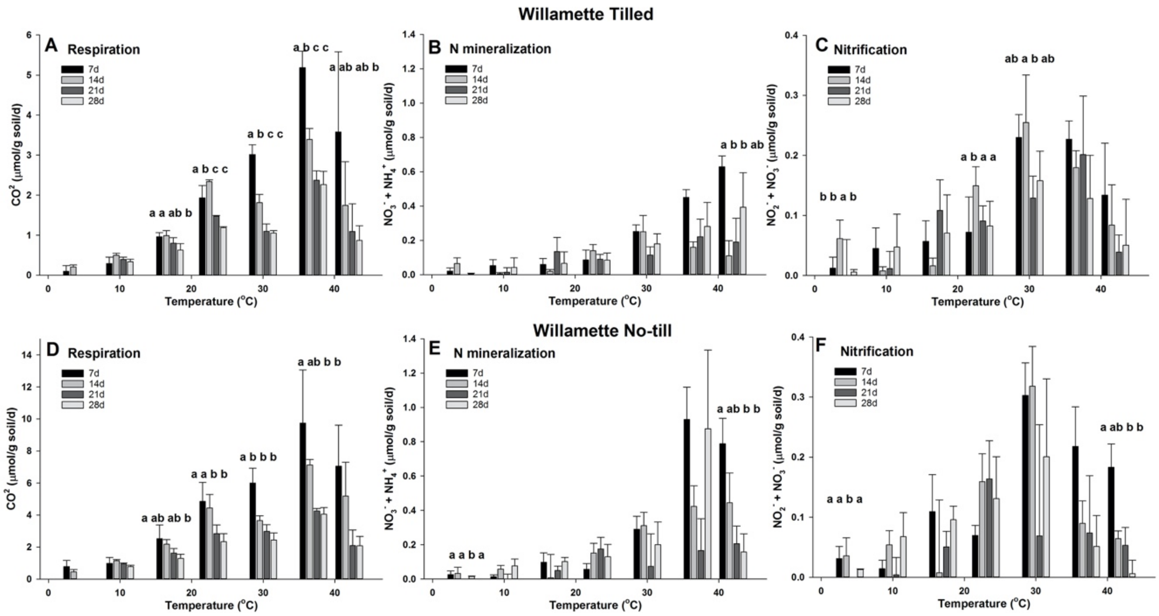

Supplemental Figure 2. The rate and thermodynamic response of respiration, N mineralization and nitrification in Willamette Valley soils across temperatures. Figure data are the average of three field replicates and the error bars represent the standard deviation. Lower case letters indicate where RM ANOVA analysis found significant differences ($p \leq 0.05$) in rates over the temperatures within a time period.

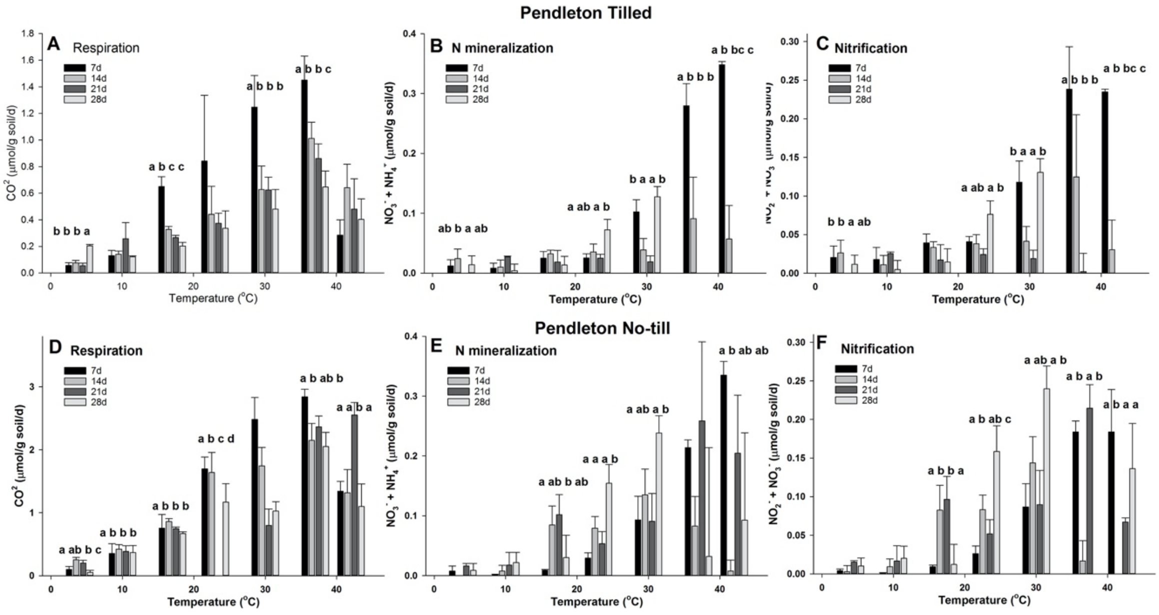

Supplemental Figure 3. The rate and thermodynamic response of respiration, N mineralization and nitrification in Pendleton soils across temperatures. Figure data are the average of three field replicates and the error bars represent the standard deviation. Lower case letters indicate where RM ANOVA analysis found significant differences ($p \leq 0.05$) in rates over the temperatures within a time period.

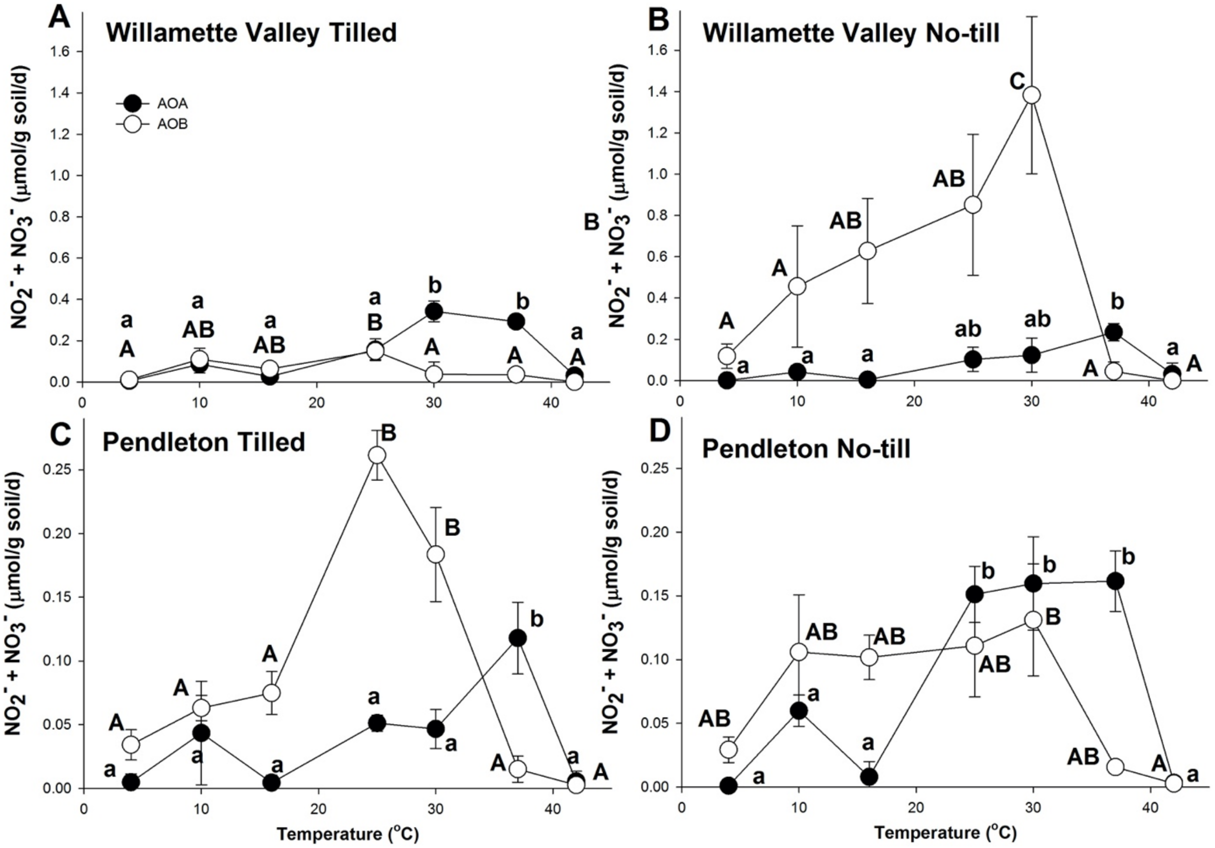

Supplemental Figure 4. Response of nitrification potential of AOA and AOB across temperature. Figure data are the average of three field replicates and the error bars represent the standard deviation. Lower case and upper case letters indicate where ANOVA analysis found significant differences in rates over the temperatures for AOA and AOB, respectively ($p \leq 0.05$).

Supplemental Table 3. Comparison of the rates accumulation of CO_2 in laboratory incubations and the modeled outcome utilizing Equation 4 and values of L_o, k and R from Supplemental Table 1. Values represent p-values from a 2-tailed T-test assuming equal variance. Comparisons where there was a significant difference between observed and estimated values ($p < 0.05$) are in bold text.

		Rate of CO_2 accumulation (µmol N/g soil/d)			
Soil	Temperature (°C)	Week 1	Week 2	Week 3	Week 4
WT	10	0.318	**0.039**	0.649	0.688
	17	0.455	0.367	0.872	0.698
	23	0.329	**0.003**	**0.012**	**0.004**
	30	0.959	0.729	0.423	0.365
	37	0.968	0.726	0.511	0.773
	42	1.000	0.997	0.962	0.942
WNT	10	0.653	0.132	0.385	0.237
	17	0.915	0.644	0.942	0.639
	23	0.837	0.501	0.633	0.872
	30	0.997	0.939	0.754	0.831
	37	0.479	0.072	0.233	0.371
	42	0.816	0.417	0.385	0.823
PT	10	0.320	0.387	0.295	0.134
	17	**0.008**	**0.033**	**0.004**	**0.001**
	23	0.349	0.778	0.429	0.353
	30	0.028	0.337	0.164	0.051
	37	0.110	0.466	0.090	**0.013**
	42	0.101	0.214	0.917	0.595
PNT	10	0.860	0.372	0.819	0.663
	17	0.390	0.188	0.184	0.446
	23	0.240	0.544	**0.001**	0.058
	30	0.321	0.407	0.633	0.455
	37	0.188	0.103	0.218	0.168
	42	**0.041**	0.268	**0.001**	0.107

Supplemental Table 4. Comparison of the rates of inorganic N accumulation in laboratory incubations and the modeled outcome utilizing Equation 5 and values of L_o, k and R from Supplemental Table 2. Values represent p-values from a 2-tailed t-test assuming equal variance. Comparisons where there was a significant difference between observed and estimated values ($p < 0.05$) are in bold text.

		Rate of inorganic N accumulation (µmol N/g soil/d)			
Soil	Temperature (°C)	Week 1	Week 2	Week 3	Week 4
WT	10	0.531	0.050	0.287	0.886
	17	0.624	**0.002**	0.248	0.917
	23	0.724	0.251	0.720	0.647
	30	0.379	0.610	0.056	0.446
	37	**0.007**	**0.004**	0.312	0.906
	42	**0.003**	**0.013**	0.134	0.701
WNT	10	**0.034**	0.245	0.069	0.190
	17	0.353	0.480	0.344	0.119
	23	0.106	0.628	0.389	0.998
	30	0.783	0.132	0.361	0.755
	37	0.056	0.584	0.130	0.224
	42	0.971	0.814	0.517	0.883
PT	10	0.169	0.396	**0.002**	0.124
	17	0.723	0.376	0.484	0.192
	23	**0.031**	0.587	**0.025**	**0.034**
	30	0.425	0.054	**0.002**	**0.007**
	37	**0.002**	0.532	**0.004**	**0.003**
	42	**0.000**	0.122	**0.001**	**0.004**
PNT	10	**0.003**	0.421	0.728	0.431
	17	**0.014**	0.259	0.112	0.340
	23	**0.006**	0.994	0.141	**0.019**
	30	0.140	0.878	0.166	**0.007**
	37	**0.010**	0.058	0.272	0.289
	42	**0.000**	**0.000**	0.564	0.416

Supplemental Table 5. Comparison of the accumulation of CO_2 in laboratory incubations and the modeled outcome utilizing Equation 6 and values of L_o, k and R from Supplemental Table 1. Values represent p-values from a 2-tailed t-test assuming equal variance. Comparisons where there was a significant difference between observed and estimated values ($p < 0.05$) are in bold text.

Accumulation of CO_2 ***(µmol/g soil)***

Soil	Temperature (°C)	Week 1	Week 2	Week 3	Week 4
WT	10	0.826	0.223	0.300	0.536
	17	0.067	0.257	0.553	0.880
	23	0.465	0.193	0.486	0.956
	30	0.162	0.086	0.076	0.094
	37	**0.045**	0.039	**0.044**	0.084
	42	0.271	0.320	0.349	0.374
WNT	10	0.143	0.165	0.190	0.256
	17	0.437	0.654	0.811	0.934
	23	0.736	0.863	0.882	0.787
	30	0.501	0.295	0.278	0.293
	37	0.875	0.826	0.885	0.858
	42	0.271	0.246	0.149	0.143
PT	10	**0.016**	0.146	0.161	0.342
	17	**0.004**	**0.025**	0.315	0.439
	23	0.416	0.641	0.871	0.931
	30	0.055	0.204	0.522	0.506
	37	**0.018**	0.077	0.311	0.988
	42	0.065	0.060	0.209	0.405
PNT	10	0.908	0.679	0.615	0.670
	17	0.883	0.907	0.646	0.424
	23	**0.001**	**0.005**	**0.004**	**0.009**
	30	**0.039**	**0.003**	0.378	0.324
	37	0.477	0.238	0.215	0.191
	42	0.274	0.931	**0.001**	0.075

Supplemental Table 6. Comparison of the accumulation of inorganic N in laboratory incubations and the modeled outcome utilizing Equation 7 and values of L_o, k and R from Supplemental Table 2. Values represent p-values from a 2-tailed t-test assuming equal variance. Comparisons where there was a significant difference between observed and estimated values ($p < 0.05$) are in bold text.

		Accumulation of inorganic N (µmol/g soil)			
Soil	Temperature (°C)	Week 1	Week 2	Week 3	Week 4
WT	10	0.117	0.295	0.681	0.668
	17	0.099	0.810	0.185	0.512
	23	0.342	0.292	0.434	0.623
	30	**0.036**	0.203	0.723	0.948
	37	**0.014**	0.531	0.596	0.688
	42	**0.000**	0.458	0.380	0.707
WNT	10	0.081	0.120	0.433	**0.007**
	17	**0.043**	0.171	0.379	0.160
	23	0.484	0.562	0.430	0.569
	30	0.975	0.660	0.684	0.702
	37	0.116	0.457	0.435	0.487
	42	0.056	0.154	0.101	**0.036**
PT	10	0.610	0.788	0.468	0.840
	17	0.237	0.220	0.210	0.662
	23	0.356	0.573	0.907	0.323
	30	0.953	0.701	0.314	0.743
	37	**0.000**	**0.013**	0.229	0.091
	42	**0.000**	**0.022**	0.343	0.090
PNT	10	**0.010**	**0.019**	0.172	0.087
	17	0.497	0.544	0.190	0.572
	23	0.683	0.816	0.393	0.397
	30	0.636	0.652	0.450	0.379
	37	**0.002**	0.561	0.057	0.943
	42	**0.000**	**0.040**	0.170	0.780

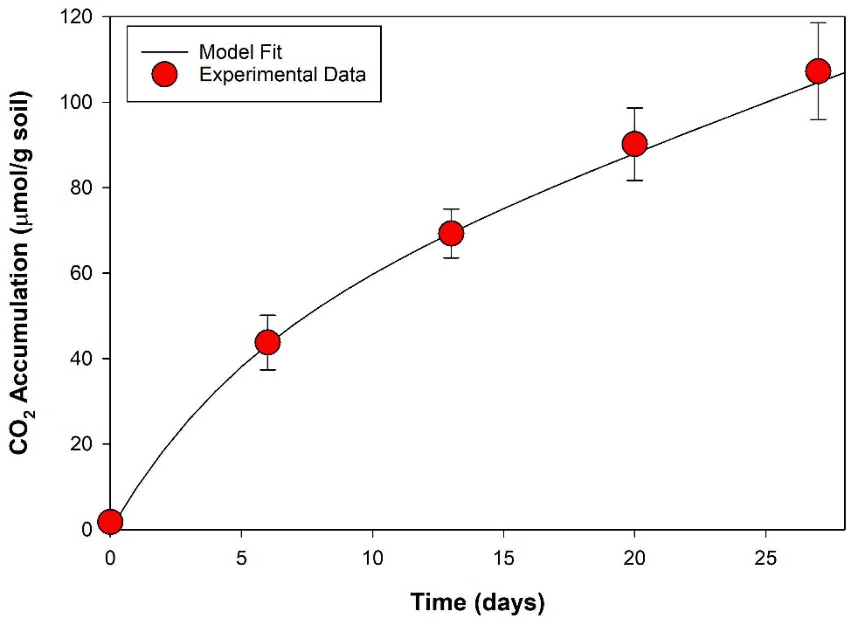

Supplemental Figure 5. Model fit of CO_2 accumulation. Example of model fit using Equation (6) and Willamette No Till soil data at 30°C. Parameter values were determined as described in Materials and Methods.

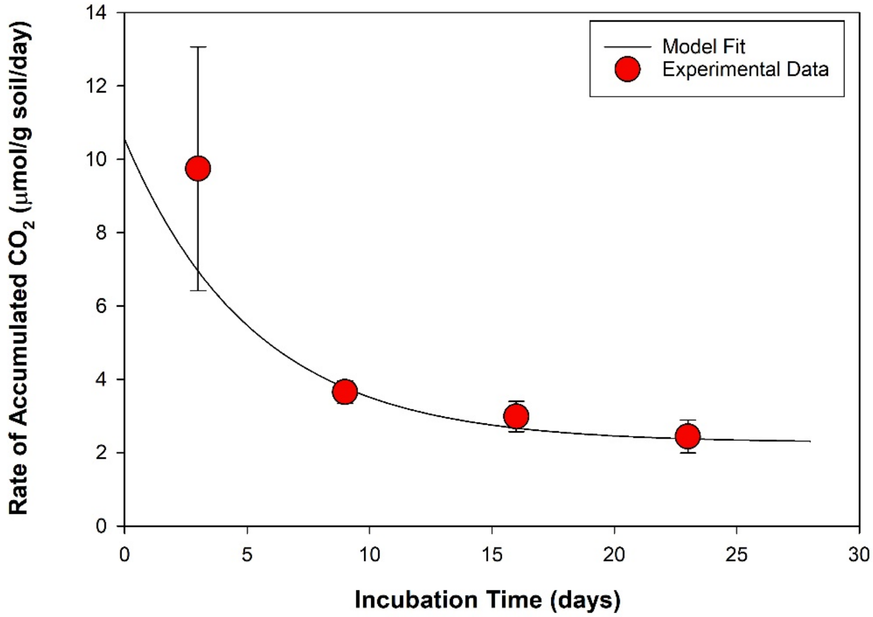

Supplemental Figure 6. Model fit of CO_2 respiration rates. Example of model fit using Equation (7) and Willamette No Till soil data at 30°C. Parameter values were determined using the integrated form of Equation (6) as described in Materials and Methods. Experimental data is an approximation of the rate at the central point of the time interval calculated as $(CO_{2\ \text{accumulation}\ (t2)} - CO_{2\ \text{accumulation}\ (t1)})/(t_2 - t_1)$ using the data shown in **Figure 5**.
